# Spatial disparities and determinants of modern contraceptive use among reproductive age women in Ethiopia: application of multilevel spatial analysis

**DOI:** 10.3389/fgwh.2025.1505789

**Published:** 2025-04-23

**Authors:** Bisrategebriel Tesfaye Muchie, Ashenafi Abate Woya, Awoke Seyoum Tegegne, Maru Zewdu Kassie, Muluhabt Alene Assfaw, Wondaya Fenta Zewdia

**Affiliations:** ^1^ Department of Statistics, Bahir Dar University, Bahir Dar, Ethiopia; ^2^ Department of Statistics, Assosa University, Assosa, Ethiopia

**Keywords:** modern contraceptive use, women, multilevel analysis, spatial analysis, Ethiopia

## Abstract

**Background:**

Contraception aims to prevent unintended pregnancies, significantly impacting maternal and infant mortality in sub-Saharan Africa, especially in Ethiopia. This study investigates factors influencing modern contraceptive use among reproductive-age women in Ethiopia.

**Methods:**

We analyzed the 2019 Ethiopia Mini Demographic and Health Survey (EMDHS) dataset, which includes 8,196 weighted samples of women and girls aged 15–49. Data management utilized STATA version 17, R version 4.2.2, and Arc GIS 10.8 for mapping. We employed multilevel and spatial analyses to identify determinants.

**Results:**

Only 26% of the women used modern contraceptives, with notable spatial clustering (Global Moran's Index = 0.237776, *p* < 0.001). Hotspots were identified in Benishangul-Gumuz; Gambela; Southern Nations, Nationalities, and Peoples’ Region (SNNPR); and eastern Oromia. Being in the 25–34 age group [adjusted hazard ratio (AHR) = 1.346, 95% CI: 1.143, 1.585]; having a higher [adjusted odds ratio (AOR) = 1.919, 95% CI: 1.380, 2.669], secondary (AOR = 1.554, 95% CI: 1.261, 1.914), or primary education level (AOR: 1.3514, 95% CI: 1.1624, 1.5712); being married (AOR = 25.953, 95% CI: 20.397, 32.942); and higher community wealth (AOR = 1.497, 95% CI: 1.114, 2.011) were positively associated with contraceptive usage, whereas being aged 35–49 (AOR = 0.538, 95% CI: 0.446, 0.649), having three or more children (AOR = 0.634, 95% CI: 0.460, 0.872), and living in the Somali region (AOR = 0.114, 95% CI: 0.045, 0.2882) were negatively associated with contraceptive usage. The best-fitting model included individual, community, and spatial variables, with an intra-class correlation coefficient indicating that 15.57% of the variability in contraceptive use was due to cluster differences.

**Conclusions:**

Modern contraceptive use among Ethiopian women is low and varies by cluster. Factors positively associated include age of 25–34, education, marital status, and community wealth. Conversely, women aged 35–49, those with three or more children, and those in the Somali region showed lower usage. Community-level interventions are necessary to improve modern contraceptive adoption.

## Introduction

Currently, family planning services support people in making decisions about whether to have children by teaching, advocating, and offering birth control methods (1). In developing countries, contraceptives have a clear effect on the health of women, children, and families (2). Modern contraceptives also make a huge contribution to the achievement of universal primary schooling, female empowerment, and reducing poverty and hunger (3). Family planning is also important in preventing unintended pregnancies and unsafe abortions (4). Unintended pregnancy is the outcome of non-use, misuse, or failure of modern contraceptive methods. It is also a major source of maternal and infant mortality rates (4).

In 2012, over 85 million, or 40%, of pregnancies worldwide were unplanned. This high number has significant consequences for the health of the mother and child, as well as the burden on the healthcare system (5). In East Africa, where maternal and child mortality is very high, the use of modern contraception is below 30% (6). The number of contraceptive-related maternal deaths is growing in developing countries, including Ethiopia (7). Since 2000, Ethiopia has seen a steady increase in modern contraceptive use. However, this increase did not result in a proportional decline in unintended pregnancy, total fertility rates, or rapid population growth (8). More prominently, target 3.7 of the Sustainable Development Goals (SDGs) highlights “the universal access to sexual and reproductive health-care services, including family planning” (5). Women may have barriers that prevent them from using contraception, although they have a strong desire to delay or limit their pregnancies. This considerable gap between women's reproductive desire and current contraceptive use is called an unmet need for family planning (6). In third-world countries, more than 200 million women who want to avoid pregnancy are not using modern contraceptive methods (9). In Ethiopia, 22% of married women have an unmet need for family planning (10). The trend of contraceptive usage varies from one region to another and may be associated with individual, societal, socioeconomic, demographic, health service-related, and cultural factors (11, 12).

Even though the prevalence of maternal mortality is substantially increasing in the study area, to the best of our knowledge, there is relatively limited research conducted in Ethiopia on modern contraceptive usage using robust statistical models (spatial and multilevel analyses). Few researchers have explored differences in contraceptive utilization to enhance family planning interventions with different methodologies in Ethiopia (13–15). However, there are barriers and gaps in understanding regarding the spatial effects of contraceptive use and this resulted in a modest increase in contraceptive use without significant changes in child and maternal mortality, fertility, and population growth. This study utilized geo-referenced data from the Ethiopia Mini Demographic and Health Survey (EMDHS), allowing us to explore the geographical variation in modern contraceptive use across zones in Ethiopia. Notably, many previous studies have used enumeration areas (EAs) as the second level in their multilevel models (16–19), while our study takes a novel approach by using zones instead. This change is crucial because enumeration areas may not be familiar to or easily understood by stakeholders, policymakers, or the general public, which limits their ability to take action in high-risk areas.

The effects of zonal variations in the country have policy implications at lower levels and are important for the preparation of health-related education for health practitioners. Using zones as administrative units enables a more precise identification of root causes and at-risk and non-risk groups, and allows us to present these areas more effectively to the relevant authorities. Furthermore, this study added community factors, such as education, wealth index, and media access, and spatial factors to the new approach of zones as the unit of analysis across Ethiopia. Understanding the key sociodemographic and reproductive factors influencing modern contraceptive use is crucial for designing effective family planning initiatives (20–22). The findings obtained in this study may be helpful to assess culturally acceptable interventions to increase modern contraceptive use. Therefore, the main aim of this study was to investigate the spatial disparities and associated factors with contraceptive usage among reproductive-age women in Ethiopia.

## Methods and participants

### Data and study setting

The data used in the current study were obtained from the 2019 EMDHS. The data were collected from 21 March 2019 to 28 June 2019 from nine geographical regions and two administrative cities in Ethiopia (23). The data were available as secondary data from the Ethiopian Mini Demographic Health Survey 2019, downloaded directly from the following free link: https://dhsprogram.com/data/. The 2019 EMDHS sample was developed to provide estimates of key indicators for the country as a whole, for urban and rural areas separately, for each of the nine regions, and for the two administrative cities. A nationally representative sample was selected using a stratified, two-phase cluster sampling technique. Based on the 2019 Ethiopia Population and Housing Census (EPHC) framework, 305 EAs were chosen in the first phase with odds proportional to their size (93 in urban areas and 212 in rural areas). In the second stage of selection, a fixed number of 30 households per cluster were selected from the newly created household list using an equal probability systematic selection method. EAs were the sampling units utilized in the first sampling phase. Ultimately, a total of 8,196 women and girls aged 15–49 were included in this study.

### Inclusion and exclusion criteria

The inclusion criteria for the current study involved Ethiopian women and girls between the ages of 15 and 49 who were non-pregnant and permanent residents of their household or visitors who stayed in the household immediately before the survey. Girls younger than 15, women older than 49, and those who were pregnant were excluded from the study.

### Variables

#### Dependent variable

The dependent/response variable was modern contraceptive utilization. If a woman used at least one of the modern contraceptive methods, she was considered a modern contraceptive user, otherwise, she was considered a non-user.

The independent variables for modern contraceptive utilization were based on previous related studies in the literature and the availability of the variable in the 2019 EMDHS dataset. Variables were broadly classified into two main groups, i.e., individual-level and community-level variables, and aligned for a multilevel analytic approach (6, 7, 24–27).

#### Individual level variables

The sex of the household head, the age of the woman, the education level of the woman, marital status, media exposure, the number of family members, the number of children under 5 in the household, religion, household wealth index, place of residence, region, and spatial auto-covariate were included as individual-level variables.

#### Community level variables

The community-level variables considered in the study include women's educational status by zone, the wealth index by zone, and media exposure by zone.

### Methods of data analysis

A weighted sample of 8,196 mothers was included in this study. Before fitting the model, exploratory data analyses were performed. Graphic and spatial analyses and a chi-square test of association were carried out to explore the relationship between the outcome variables and each independent variable.

### Spatial analysis

Spatial data analysis is an analysis in which geographical locations are taken into account. The primary feature of spatial statistical models is that nearby attribute values are more statistically dependent than distant attribute values (28). Spatial dependence occurs when values at one location depend on neighboring observations, leading to unique analysis approaches. Proximity between zones impacts relationships, known as spatial autocorrelation, influencing spatial prediction methods based on regionalized variable theory (29).

### Spatial weight matrix

In the current study, the spatial weight matrix, $W_{ij}$, was used to describe the structure of neighboring or spatial structures among locations, with every unit area described as rows and columns, and these weights were referred to as neighboring functions (30, 31).

### Spatial autocorrelation

In this study, spatial autocorrelation was also used to measure the relationships among nearby values of $x_{i}$ where the meaning of “nearby” is specified by $W_{ij}\;$(weighted matrix), but a more useful spatial autocorrelation statistic, called Moran's I, can be produced by standardizing the spatial auto-covariance. A global measure of spatial correlation was used to investigate whether the contraceptive usage distribution was the same pattern or process that occurs over the entire geographic area and calculate an average for the entire area.

The value of Moran's I ranges from −1 to 1. The value can be interpreted similarly to correlation coefficients. When the neighboring regions have similar values, the value of Moran's I will be positive, and when the neighboring regions have dissimilar values, Moran's I will be negative (32).

### Hot and cold spot analysis

Hot and cold spot analysis was used to calculate the Getis–Ord, Gi *, statistic for each feature in a dataset. Hot spot analysis (the Getis–Ord Gi* statistic) of the z-scores and significant *p*-values indicates the features with either hot spot or cold spot values for the spatial clusters spatially (33).

### Spatial interpolation and Kriging

Spatial interpolation was used to predict values in a certain area using observed data through a method known as interpolation. In this study, ordinary Kriging interpolation was used to estimate the prevalence of contraceptive usage at non-sampled locations in Ethiopia. To minimize prediction uncertainty and filter out measurement errors, the Ordinary Kriging Gaussian interpolation technique was also used (34).

### Multilevel binary logistic regression (two-level model)

Multilevel analysis is a statistical technique that extends ordinary regression analysis to situations where the data are multilevel or hierarchical, correcting for biases in parameter estimates resulting from clustering (35). In hierarchical datasets such as the EMDHS, individuals within clusters share characteristics, leading to intra-cluster correlation. Ignoring this structure violates the independence assumption, causing biased estimates and incorrect inferences. To address this, a two-level mixed-effects logistic regression model was employed to analyze modern contraceptive utilization, incorporating both individual-level and community-level fixed effects and accounting for between-cluster variability through random effects. The multilevel binary two-level model is represented as follows:

(1) $$\mathrm{logit}(\pi_{ij})=\log\left(\frac{\pi_{ij}}{1-\pi_{ij}}\right)=\;\beta_{0j}+\;\beta_{kj}X_{kij}+\Upsilon_{0q}Z_{qj\;.}$$

where $\beta_{0j}\;$ is the average intercept $\Upsilon_{00}\;$ plus group-dependent deviation $U_{0j}:$, which is $\beta_{0j}=\Upsilon_{00}+U_{0j}$ and $\beta_{kj}=\Upsilon_{k0}+U_{kj}$. Thus, the above two-level model (Equation 1) can be rewritten more generally, containing all levels of variation in Equation 2:

(2) $$\mathrm{logit}(\pi_{ij})=\log\left(\frac{\pi_{ij}}{1-\pi_{ij}}\right)=\Upsilon_{00}+\Upsilon_{k0}X_{kij}+\Upsilon_{0q}Z_{qj}+U_{0j}+U_{kj}$$

where $\Upsilon_{00}$ is the average regression intercept, $X_{kij}$ represents the *k* level-one covariates, and $Z_{qj}\;$ represents the *q* level-two covariates, whereas, $\Upsilon_{k0}$ and $\Upsilon_{0q}$ are the corresponding regression coefficients for level-one and level-two explanatory variables, and $U_{0j}\;$ and $U_{kj}\;$ are the random effect of the model parameters at level two. Akaike's information criterion (AIC) and the Bayesian information criterion (BIC) were used to determine the best multilevel model in this study.

### Intra-class correlation

The intra-class correlation (ICC) represents the proportion of cluster-level variance compared to the total variance, indicating the extent to which the total variance is attributed to the cluster level (35). It also signifies whether there is a need for higher-level analysis. To examine random effects, we utilized the ICC and the proportional change in variance (PCV). The ICC is a measure of the similarity between two people from the same neighborhood and was a value between 0 and 1 inclusive. If there was no variation between the area effects, then ICC  = 0.

### Model selection

Once the models were fitted, the next important step was to choose the best model among the fitted models. The model selection method is the best process for finding the simplest and most well-fitted model for the data from the different proposed models. There are different methods with different model selection criteria. AIC and the BIC were used to determine the best model in this study.

## Results

**Figure 1:**
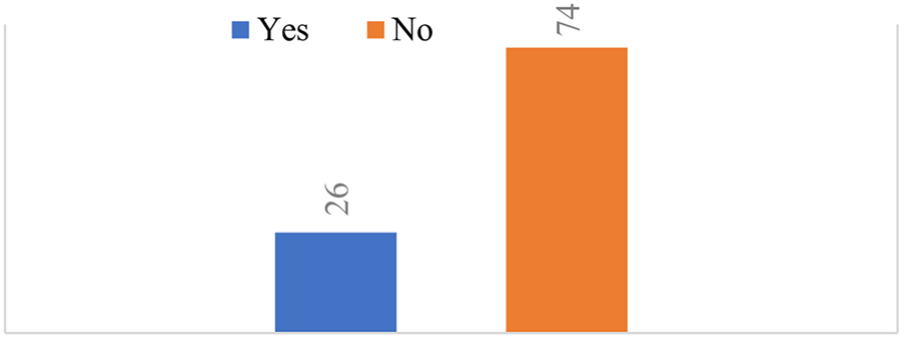
Proportion of modern contraceptive usage.

According to the proportion of modern contraceptive usage, 26% of women used modern contraceptive methods and 74% were non-users (Figure 1). The results, as shown in Table 1, revealed the chi-square association and weighted frequency of the independent variables with respect to modern contraceptive use. The separate chi-square statistics revealed that modern contraceptive use was significantly associated with predictor variables including sex of the household head, age of the woman, mother's educational level, mother's marital status, media exposure, number of family members, number of children under 5 in the household, household wealth index, and religion of respondents (*p*-value ≤ 0.25). For the sex of the household head variable, 78.5% were male and 21.5% were female. In the male-headed households, 88.8% of women were non-users of modern contraceptives. The highest prevalence (45.5%, *n* = 1,134) of modern contraceptive use was recorded among women aged 25–34 years. Mothers with a primary education accounted for 3,430 (41.8%), out of them, 1,059 (42.4%) used modern contraceptive methods. Married mothers accounted for 5,075 (61.9%) of the total, with 2,320 (93%) using modern contraceptives. Of the 3,191 respondents with media access, 1,091 (43.7%) used modern contraceptive methods. Among households with fewer than five family members, only 1,472 (59%) of those women used modern contraceptive methods. Most women in families with two or fewer children under 5 in the household (97.2%) used modern contraceptive methods. Among the wealthiest household respondents, 721 (29%) used modern methods. Based on our findings, the majority of respondents belonged to the Orthodox religion, with 46.7% using modern methods.

**Table 1:** Association between individual independent variable characteristics and the use of modern contraceptives.

Variable	Category	Weighted frequency *n* (%)	Modern contraceptive use (%)	Chi-square *p*-value
Sex of the household head	Male	6,436 (78.5)	2,215 (88.8)	<.0001
	Female	1,760 (21.5)	280 (11.2)	
Age of the woman	15–24	3,459 (42.2)	722 (28.9)	<.0001
	25–34	2,483 (30.3)	1,134 (45.5)	
	35–49	2,254 (27.5)	639 (25.6)	0.0567
Mother's educational level	No education	3,279 (40.0)	992 (39.8)	
	Primary	3,430 (41.8)	1,059 (42.4)	
	Secondary	1,031 (12.6)	286 (11.5)	
	Higher	456 (5.6)	158 (6.3)	
Mother's marital status	No married	3,121 (38.1)	175 (7.0)	<.0001
	Married	5,075 (61.9)	2,320 (93.0)	
Media exposure	No	5,005 (61.1)	1,404 (56.3)	<.0001
	Yes	3,191 (38.9)	1,091 (43.7)	
Number of family members	<=5	4,119 (50.3)	1,472 (59.0)	<.0001
	Six and above	4,077 (49.7)	1,023 (41.0)	
Number of children under 5 in household	Two or less	7,819 (95.4)	2,426 (97.2)	
	Three and above	377 (4.6)	69 (2.8)	<.0001
Religion of respondents	Orthodox	3,473 (42.4)	1,166 (46.7)	
	Catholic	46 (0.5)	21 (0.8)	
	Protestant	2,239 (27.3)	761 (30.5)	
	Muslim	2,361 (28.8)	526 (21.1)	
	Tradition	64 (0.8)	17 (0.7)	
	Other	13 (0.2)	4 (0.2)	<.0001
Household wealth index	Poorest	1,264 (15.4)	290 (11.6)	
	Poor	1,491 (18.2)	415 (16.6)	
	Middle	1,544 (18.9)	530 (21.2)	
	Richer	1,766 (21.5)	539 (21.6)	
	Richest	2,131 (26.0)	721 (29.0)	

### Community-level characteristics description

Most of the respondents lived in rural areas, accounting for 5,523 (67.4%) of the total sample. Among them, 1,653 (66.3%) reported using modern contraceptive methods. Furthermore, 3,070 respondents were from the Oromia region, of which 919 (36.8%) utilized modern methods. Additionally, 4,452 (54.3%) women had attained higher education, and among them, 59% used modern contraceptive methods. Regarding the wealth index, 4,185 (51.1%) women were classified as rich, with 56% of this group using modern methods. In total, 4,199 (51.2%) women had community-level media access, with 1,415 (56.7%) of those individuals being modern method users (Table 2).

**Table 2:** Community-level variable frequency.

Variable	Category	Weighted frequency *n* (%)	Modern contraceptive use (%)	Chi-square *p*-value
Place of residence	Urban	2,673 (32.6)	842 (33.7)	0.1493
	Rural	5,523 (67.4)	1,653 (66.3)	
Region	Tigray	578 (7.0)	165 (6.6)	<.0001
	Afar	76 (0.9)	10 (0.4)	
	Amhara	1,915 (23.4)	697 (27.9)	
	Oromia	3,070 (37.5)	919 (36.8)	
	Somali	362 (4.4)	12 (0.5)	
	Benishangul	90 (1.1)	26 (1.0)	
	SNNPR	1,574 (19.2)	531 (21.3)	
	Gambela	37 (0.5)	9 (0.4)	
	Harari	24 (0.3)	5 (0.2)	
	Addis Ababa	411 (5.0)	109 (4.4)	
	Dire Dawa	59 (0.7)	12 (0.5)	
Community education level	Lower	3,744 (45.7)	1,014 (40.6)	<.0001
	Higher	4,452 (54.3)	1,481 (59.4)	
Community wealth index	Lower	4,011 (48.9)	1,099 (44.0)	<.0001
	Higher	4,185 (51.1)	1,396 (56.0)	
Community-level media access	Lower	3,997 (48.8)	1,080 (43.3)	<.0001
	Higher	4,199 (51.2)	1,415 (56.7)	

SNNPR: Southern Nations, Nationalities, and People's Region.

### Spatial analysis

#### Spatial autocorrelation analysis of modern contraceptive use

**Figure 2:**
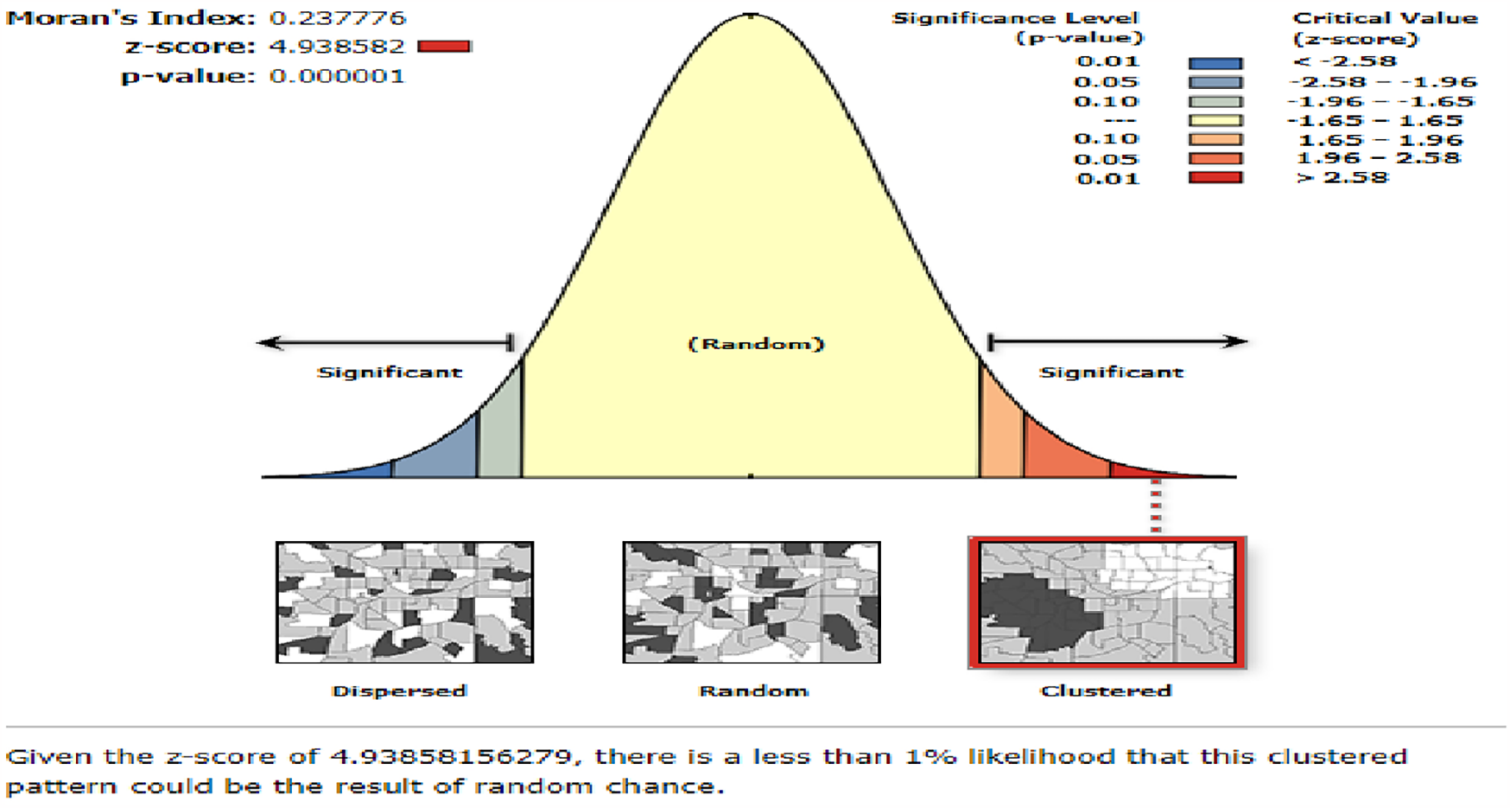
Spatial autocorrelation analysis use of modern contraceptives among women in Ethiopia from the 2019 Ethiopia Mini Demographic and Health Survey.

Using Moran's I index, one can detect whether or not the use of modern contraceptives in a given cluster is similar to that of neighboring clusters. The estimated global Moran's I in this study was 0.237776, which indicates that the spatial distribution of the use of modern contraceptives was significantly clustered across zones in Ethiopia. The Moran's I *p*-values for the use of modern contraceptives were both less than 0.05 (Figure 2).

#### Spatial distribution of use of modern contraceptive

**Figure 3:**
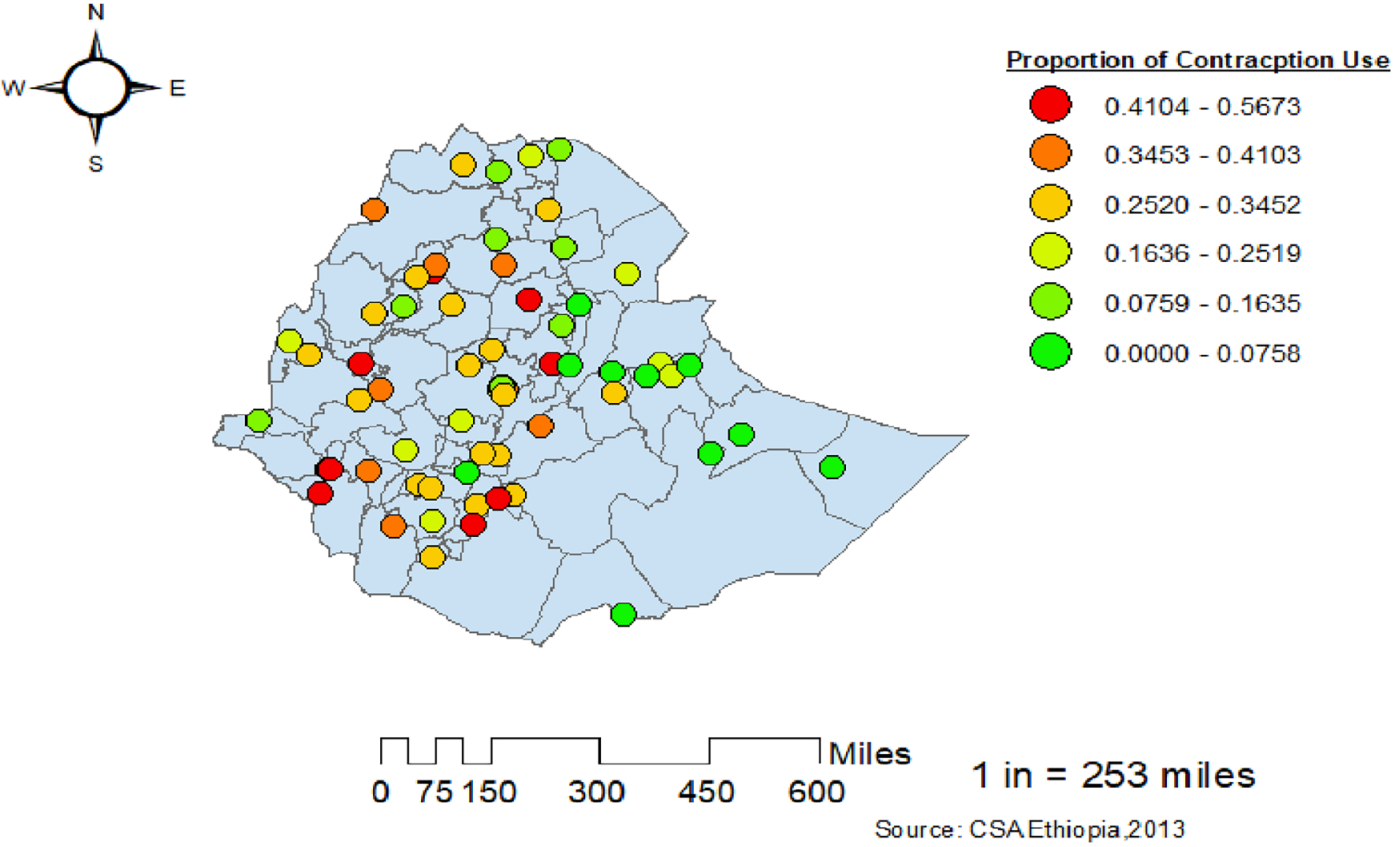
Spatial distribution of use of modern contraceptives among women in Ethiopia from the 2019 Ethiopia Mini Demographic and Health Survey.

A total of 64 administrative zones were included in this study to observe the spatial distribution of the use of modern contraceptives in Ethiopia. Each point on the map characterizes the proportion of women who used modern contraceptives per cluster. The red color indicates the highest proportion of modern contraceptive use among women in Ethiopia, while the green color represents the lowest proportion, predominantly in the eastern part of the country (Figure 3).

#### Hot spot analysis of women’s use of modern contraceptives

**Figure 4:**
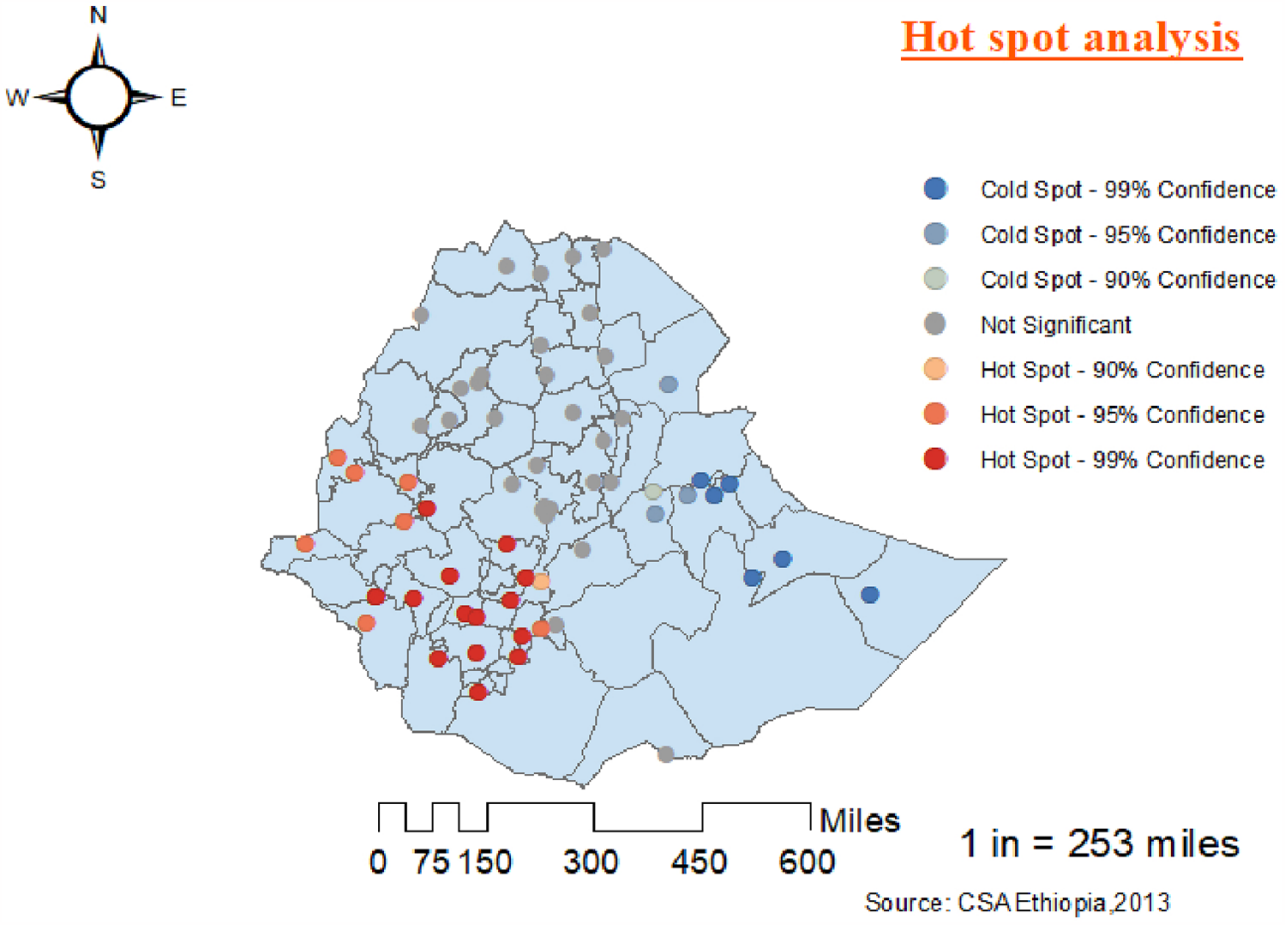
Hot spot and cold spot analysis of the use of modern contraceptives among women in Ethiopia from the 2019 Ethiopia Mini Demographic and Health Survey.

According to the local Getis–Ord Gi* statistics, there were significant hot and cold spot areas for the use of modern contraceptive methods among women. The red color indicates significant hot spot (high-risk) areas for the use of modern contraceptives by women, while the bright blue indicates cold spot (low-risk) areas. The western parts of the country showed high usage of modern contraceptives among women, whereas the eastern parts showed lower usage (Figure 4).

#### Spatial interpolation of women’s use of modern contraceptive

**Figure 5:**
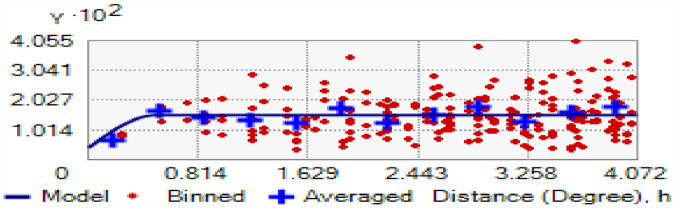
Semi-variogram for the use of modern contraceptive women from the 2019 Ethiopia Mini Demographic and Health Survey.

Based on mean square error (MSE), the spherical model was better (unbiased prediction) with a closer value to zero than the others, even though the difference was small. This spherical model resembles the relationships between sample points that decay gradually (Figure 5). As shown in Table 3, the nugget-to-sill ratio for modern contraceptive use among women was 0.27, reflecting a spatial autocorrelation of 27%. This ratio represents the degree of spatial dependency, indicating that there was a spatial correlation in the use of modern contraceptives.

**Table 3:** Comparison of various semi-variogram models and their parameters.

Model	Circular	Spherical	Exponential	Gaussian
MSE	0.1271	0.1270	0.128	0.128
RMSE	0.996	0.998	1.01	0.9998
Parameters for the selected model (spherical model)				
Nugget = 0.0041				
Sill = 0.0151				
Nugget-to-sill ratio (%) = 0.27 (27%)				

MSE, mean square error; RMSE, root mean square error.

The spatial Kriging interpolation analysis was used to predict the use of modern contraceptives among women for non-sampled areas of the country based on the given measurements. The predicted high-risk and low-risk areas for the use of modern contraceptives among women are indicated by the red and bright blue colors, respectively. The predicted use of modern contraceptives among women in the area increases from the green to the red-colored areas.

**Figure 6:**
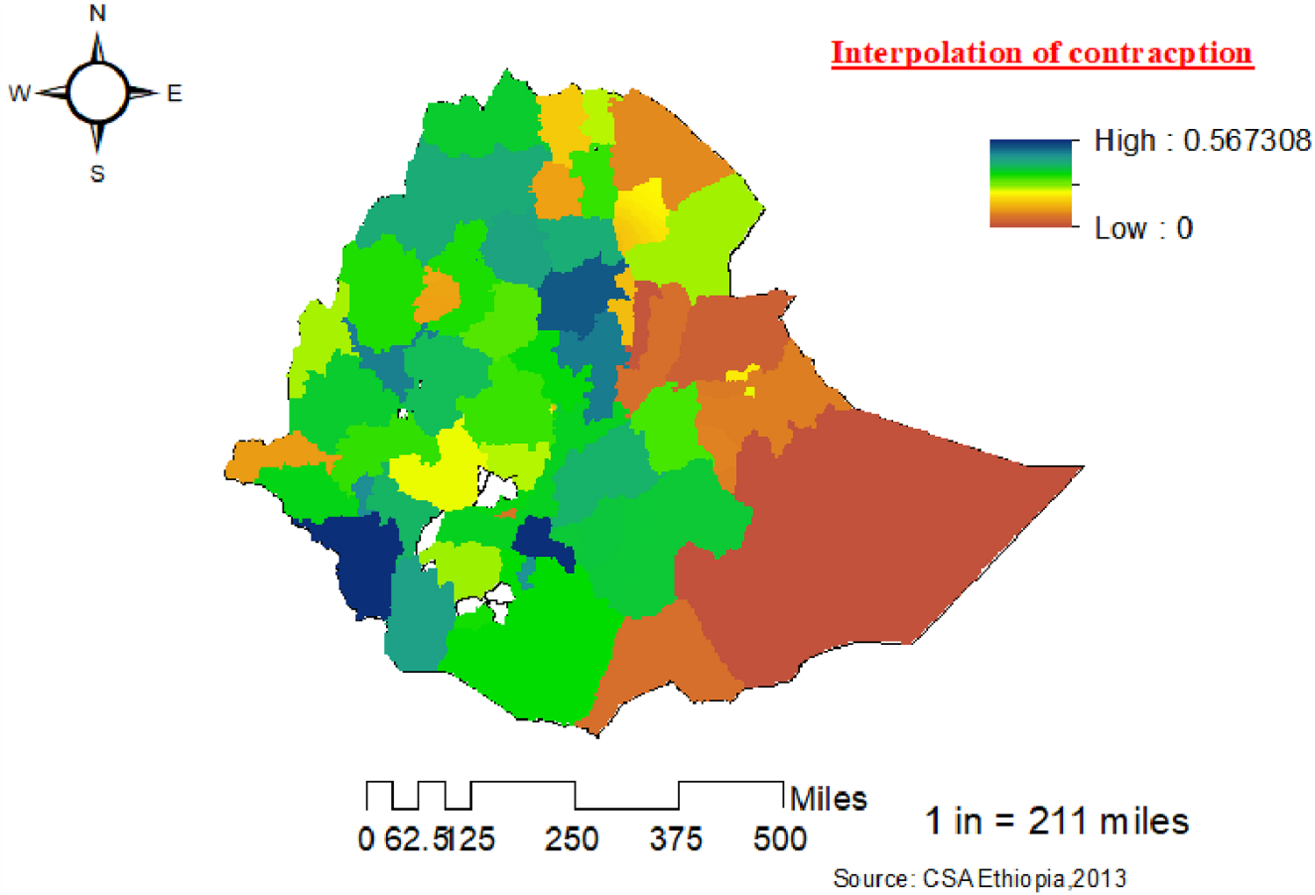
Kriging interpolation of the use of modern contraceptives among women in zones in Ethiopia from the 2019 Ethiopia Mini Demographic and Health Survey.

The red color indicates high-risk areas for the predicted use of modern contraceptives among women. The Somali, Afar, and Dire Dawa regions and the eastern parts of the Oromia region have been identified as high-risk areas for modern contraceptive use compared to other regions. The bright blue color indicates the predicted low-risk areas for the use of modern contraceptives, which are located in the Addis Ababa, Amhara, Gambela, south-west Oromia, north-east Tigray, and Benishangul zones (Figure 6).

### Model fitting and parameter estimation

#### Random effect analysis results

In this study, the outcome variable was modern contraceptive usage, which is a binary response. To account for the variation of the outcomes across the clusters, multilevel logistic regression models were fitted.

#### Model -I: intercept-only model

The intercept-only model without an explanatory variable was constructed to measure the effect of community variation on modern contraceptive use. The variance of the random effects at the cluster level was $\sigma_{u0\;}^{2}$= 0.6068 (*p*-value < 0.0001), which was statistically significant and reflects there was statistically significant variation in modern contraceptive use across the community (Table 4). The estimated intra-class correlation was computed as ICC = $\sigma_{u0\;}^{2}/(\sigma_{u0}^{2}+3.29)$ = 0.6068/(0.6068+3.29) = 0.1557 = 15.57%. This indicates that 15.57% of the total variation for modern contraceptive use was due to the difference between communities, leaving 84.43% of the variability to be accounted for by factors related to the women or other unknown factors. Moreover, a PCV of 0.24 indicated that 24% of the total variation in contraceptive usage was explained by the full model (model IV)**.** The result of the multilevel logistic regression model considering different level 1 and level 2 effects is presented in Table 4 and the most well-fitted model was selected based on the lowest AIC and BIC values. The model that included individual-level, community-level, and spatial auto covariate variables (model-IV) was the best-fitted model for the data based on the lower AIC and BIC fit statistics compared to the other models (Table 4). Therefore, a multilevel generalized linear mixed model (GLMM) that accounts for spatial effects was the most parsimonious model for the data (Table 4).

**Table 4:** Model selection.

Parameter	Model I	Model II	Model III	Model IV
Variance	0.6068	0.5951	0.4740	0.4642
ICC	Ref.			0.1557
PCV	Ref.			0.24
AIC (smaller is better)	9,549.60	7,391.05	7,325.80	7,317.71
BIC (smaller is better)	9,557.04	7,469.17	7,456.01	7,431.64

ICC, intra-class correlation; PCV, proportional change in variance; AIC, Akaike information criterion; BIC, Bayesian information criterion.

As presented in Table 5 below, the results of the multilevel GLMM show that contextual region, community-level education, community-level wealth index, age of the woman, mother's education, marital status, the number of children under 5 in the household, wealth index, and religion had significant effects (p-value<0.05) on the log-odds probability of the *i*th woman in the *j*th cluster using modern contraceptive methods. Since the dependent variable, modern contraceptive use, has two categories, the intercept was estimated at −4.7662. However, we were more interested in interpreting the estimated probability of women in cluster *j* falling into the category of “modern contraceptive use = Yes.” More importantly, our study showed that the use of modern contraceptive methods was positively correlated with women aged 25–34, married, educated, and residing in the Amhara region, as well as those with higher community education. In contrast, the use of contraceptives was negatively correlated with being 35–49 years old, having three or more children, and residing in the Somali region.

**Table 5:** Parameter estimation.

Variable (category)	Estimate (SE)	95% CI	Odds ratio
Intercept	−4.7662 (0.7983)***	(−6.3374, −3.1950)	0.009
Sex of the household head			
Female	−0.03995 (0.1003)	(−0.2373, 0.1574)	0.961
Male	Ref		
Age of the woman			
25–34	0.2971 (0.08350)**	(0.1331, 0.4611)	1.346
35–49	−0.6196 (0.09564)**	(−0.8074, −0.4318)	0.538
15–24	Ref.		
Mother's educational level			
Higher	0.6519 (0.1678)***	(0.3225, 0.9812)	1.919
Primary	0.3012 (0.07690)***	(0.1502, 0.4522)	1.351
Secondary	0.4407 (0.1065)**	(0.2007, 0.6807)	1.554
No education	Ref		
Mother's marital status			
Married	3.2555 (0.1222)***	(3.0458, 3.4651)	25.933
No married	Ref.		
Media exposure			
Yes	0.09762 (0.07735)	(−0.0546, 0.2499)	1.103
No	Ref.		
Number of family members			
Six and above	−0.03476 (0.06816)	(−0.1689, 0.09936)	0.966
≤5	Ref.		
Number of children under 5			
Three and above	−0.4564 (0.1632)**	(−0.7787, −0.1341)	0.634
Two or less	Ref.		
Religion of respondent			
Catholic	1.0112 (0.8353)	(−0.6330, 2.6555)	2.749
Muslim	0.2596 (0.7475)	(−1.2119, 1.7311)	1.296
Orthodox	0.7289 (0.7446)	(−0.7368, 2.1945)	2.073
Protestant	0.5935 (0.7373)	(−0.8579, 2.0449)	1.81
Tradition	0.3328 (0.8188)	(−1.2791, 1.9447)	1.395
Other	Ref.		
Household wealth index			
Poorer	0.1420 (0.1215)	(−0.09667, 0.3807)	1.153
Middle	−0.09852 (0.1155)	(−0.3254, 0.1284)	0.906
Richer	−0.2288 (0.1290)	(−0.4821, 0.02456)	0.795
Richest	0.04551 (0.1680)	(−0.2845, 0.3755)	1.047
Poorest	Ref.		
Place of residence			
Urban	0.2485 (0.2074)	(−0.1598, 0.6567)	1.282
Rural	Ref.		
Region			
Addis Ababa	−0.3305 (0.3302)	(−0.9804, 0.3193)	0.719
Afar	−0.8075 (0.4802)	(−1.7527, 0.1377)	0.446
Amhara	0.5700 (0.2708)*	(0.03710, 1.1030)	1.768
Benishangul	0.1856 (0.4284)	(−0.6576, 1.0289)	1.204
Dire Dawa	−0.5690 (0.5338)	(−1.6197, 0.4817)	0.566
Gambela	−0.3308 (0.5400)	(−1.3936, 0.7321)	0.718
Harari	−0.5566 (0.6558)	(−1.8474, 0.7341)	0.573
Oromia	0.1964 (0.2972)	(−0.3886, 0.7813)	1.217
SNNPR	0.3444 (0.3016)	(−0.2493, 0.9381)	1.411
Somalia	−2.1684 (0.4698)***	(−3.0930, −1.2438)	0.114
Tigray	Ref.		
Community education			
Higher	0.3120 (0.1550)*	(0.007058, 0.6170)	1.366
Lower	Ref.		
Community wealth index			
Higher	0.4033 (0.1507)**	(0.1067, 0.6999)	1.497
Lower	Ref.		
Community-level media access			
Higher	0.1156 (0.1690)	(−0.2170, 0.4481)	1.123
Lower	Ref.		
Si (spatial auto-covariate)	0.5948(0.4267)	(0.2348, 0.8714)	1.813

OR, odds ratio; CI, confidence interval; SNNPR, South Nations, Nationalities, and Peoples’ Representatives.

*** *p* < 0.001.

** *p* < 0.01.

* *p* < 0.05.

After adjusting for other covariates, the estimated odds of women aged 35–49 using modern contraceptive methods in the same cluster was 0.538 ($e^{-0.6196}$) times more than the estimated odds of women aged 15–24. This indicates that the estimated odds of women aged 35–49 using modern contraceptive methods were 46.2% lower than the estimated odds of women aged 15–24 in the same clusters. Notably, after adjusting for other covariates, the estimated odds of women aged 25–34 using modern contraceptive methods in the same cluster was 1.346 ($e^{0.2971}$) times more than the estimated odds of women aged 15–24. This reveals that the 25–34 age group was more likely to use modern contraceptives in Ethiopia compared to the 15–24 age group. The estimated odds of women with a higher educational level using modern contraceptive methods in the same clusters was 1.919 $\left(e^{0.6519}\right)$ times more than the estimated odds of women with no education. This indicates that the estimated odds of women with a higher educational level using modern contraceptive methods were 91.9% higher than the estimated odds of women with no education in the same clusters, keeping all other variables constant. This result indicates that as women's education levels increase, the use of modern contraceptives also increases in Ethiopia.

The odds of women living in the Amhara region using modern contraceptive methods in the same clusters were 1.768 ($e^{0.57}$) times more than the odds of women living in the Tigray region. This reflects that the odds of women living in Amhara using modern contraceptive methods were 76.8% higher than the odds of women living in Tigray regions of the same clusters, keeping all other variables constant. Additionally, the odds of married women using modern contraceptive methods in the same clusters were 25.93 times higher than those of non-married women. The odds of women with more than three children under 5 in the household using modern contraceptive methods in the same clusters were 0.63 times lower than those of women with fewer than three children under 5 in the household. As the number of family members increased, the percentage of modern contraceptive usage also increased, likely as a means to limit family size.

Moreover, the odds of women from communities with a higher wealth index using modern contraceptive methods within the same clusters were 1.49 times greater than those from poorer communities. As the community wealth index increased, both knowledge of and use of modern contraceptives also increased. This may be because highly affluent communities are more likely to have access to well-equipped healthcare facilities.

Finally, the odds of women from communities with higher education levels using modern contraceptive methods within the same clusters were 1.366 times greater than those of women with no formal education (Table 5).

## Discussion

This study investigated the use of modern contraceptives among women considering individual- and community-level variables across the administrative zones in Ethiopia. The study was evaluated using different statistical models, and the best model selected for this study, based on the AIC and BIC values, was the multilevel GLMM that accounts for spatial effects. The results of this study show that age, education level, marital status, number of children under 5, religion, community-level education, and community-level wealth index were statistically significant factors associated with modern contraceptive use across the zones and they are discussed below. This study further revealed that the use of modern contraceptive methods varied among women across the zones, as supported by previous studies (24, 25, 36). The prevalence of modern contraceptive use was 26%. This finding aligns with the report from the 2019 EMDHS in Ethiopia and was lower than that in other sub-Saharan countries (29%) (23, 37).

This study showed that women aged 25–34 were more likely to use modern contraceptives compared with women and girls aged 15–24 in Ethiopia. This important finding corroborates the findings obtained in previous studies in Ethiopia (27). A possible reason for this finding is that women aged 25–34 have a better understanding of the consequences of engaging in sexual acts without contraception compared to women and girls aged 15–24. Women and girls aged 15–24 may also have challenges accessing family planning services because they have no knowledge of where to obtain contraception or cannot afford the services (6, 26, 27, 38).

The results of this study also show that women who had attained a primary school education had higher odds of using modern contraceptives than their uneducated counterparts. This finding is consistent with previous studies that have shown a similar pattern of relationship between educational status and contraceptive use (3, 7, 9). Education empowers women to have autonomy in making important decisions regarding fertility-related issues and helps them exercise reproductive health rights compared to uneducated women. Educated women are likely to have a greater comprehension of the advantages of utilizing contraception to prevent unintended pregnancies compared to women with limited education. Therefore, it is imperative for family planning service providers to prioritize and cater to the needs of women with lower levels of education during family planning sessions. By equipping them with fundamental reproductive health information, these sessions can enhance the acceptance and utilization of contraceptives among this demographic (27, 38, 39). Moreover, it is important for policymakers in Ethiopia to formulate and enforce policies that promote the education of girls and women.

Studies have found that women who have never married are more likely to use modern contraceptives to prevent unintended pregnancies. Married individuals often face societal expectations to start a family shortly after being married, which can lead to unintended pregnancies even if they initially planned to postpone having children (6, 26, 27, 38, 39). The number of children under 5 living in the household was associated with the odds of using modern contraception in this study and similar associations were found in the DHSs of other countries. A woman with more than three children under 5 living in the household had higher odds of using modern contraceptives and the odds increased as the number of children increased. In nulliparous women, the desired number of children is unmet and the intention to bear a child is high, thus, they are less likely to use contraceptives. As the number of children increased, women tended to use contraceptives as their desired number of children was met (6, 10, 27). In the case of great-grand multiparous women, some may have been unaware of modern contraceptive methods, or in certain cases cultural preferences regarding the sex of children may have influenced their reproductive decisions.

Community-level wealth index was also found to be a significant factor influencing contraceptive use among the clusters. This factor was significant in different studies (7, 9). Economically poorer areas have worse health facilities and the distance to the health facility can be far. Poorer communities do not invest in women’s education and there is less women empowerment. Community cultural barriers may also be greater in these communities (40).

Furthermore, there were regional variations in Ethiopia's use of modern contraceptive methods. Compared to women in Tigray, women in the Somali region were less likely to use modern forms of birth control (41). In contrast, the rate of non-users was lower in the Amhara region compared to the Tigray region (10, 27, 36). A possible reason for this regional disparity is that there are differences in the implementation of family planning services across regions (13, 39). In Ethiopia, the lack of access to contraceptive methods has contributed to the country's high under-5 mortality rate. This implies that having access to contraceptive methods reduces child and infant mortality and adds to the health complications of mothers (42, 43).

### Limitations of the study

Similar to other research studies, this study has its limitations. Specific challenges include the lack of certain variables, the presence of missing values in the MDHS, and reporting and recall biases. We only considered 64 Ethiopian administrative zones because the remaining eight zones were not included in the data collection of the EMDHS 2019. Therefore, further studies should investigate contraceptive usage by incorporating all Ethiopian zones when the EDHS data is released.

### Conclusion

The finding of this study concluded that among the women included in this study, more than one-fourth (26%) used modern contraceptives in Ethiopia based on 2019 EMDHS. Additionally, the results show that modern contraceptive use varied across the 64 zones in Ethiopia. We also concluded that the distribution of modern contraceptive use was clustered, indicating that it was not random across the Ethiopian zones. High-risk (hot spot) areas for modern contraceptive use were found in Somali, Afar, and the southeastern parts of Ethiopia, while low-risk areas were identified in the central, southern, and most of the western and eastern parts of the country.

Furthermore, the study concludes that individual characteristics, socioeconomic factors, and environmental characteristics were important determinants of modern contraceptive use among women in Ethiopia. Our study also discovered that being aged 25–34, educated, and married; living in the Amhara region; and a higher community education level were positively associated with the use of modern contraceptive methods. Conversely, being aged 35–49, having three or more children, and living in the Somali region were negatively associated with contraceptive use. Traditional and cultural beliefs that encourage early marriage and childbirth may be the principal reasons for the observed geographical variation.

Therefore, the government and concerned stakeholders should reshape their contraceptive policies in high-risk zones. Future studies should explore community-level cultural beliefs and the availability of health services and include other important covariates not addressed in this study to investigate the geographical and social differences in modern contraceptive use among women, especially at lower administrative levels (woreda or kebele level).

## Data Availability

The datasets presented in this study can be found in online repositories. The names of the repository/repositories and accession number(s) can be found below: https://www.dhsprogram.com/data/dataset_admin/login_main.cfm.
